# Immunocyte Profiling Using Single-Cell Mass Cytometry Reveals EpCAM^+^ CD4^+^ T Cells Abnormal in Colon Cancer

**DOI:** 10.3389/fimmu.2019.01571

**Published:** 2019-07-09

**Authors:** Ting Zhang, Junwei Lv, Ziyang Tan, Boqian Wang, Antony R. Warden, Yiyang Li, Hui Jiang, Hao Li, Xianting Ding

**Affiliations:** ^1^State Key Laboratory of Oncogenes and Related Genes, School of Biomedical Engineering, Institute for Personalized Medicine, Shanghai Jiao Tong University, Shanghai, China; ^2^Department of General Surgery, Affiliated First People's Hospital, Shanghai Jiao Tong University, Shanghai, China

**Keywords:** colon cancer, EpCAM, T cell immunity, mass cytometry, p38 MAPK

## Abstract

Colon cancer (CC) is one of the leading causes of cancer related mortality. Research over past decades have profoundly enhanced our understanding of immunotherapy, a major clinical accomplishment, and its potential role toward treating CC. However, studies investigating the expression of these immune checkpoints, such as epithelial cell adhesion molecule (EpCAM), programmed death-1 (PD-1), and programmed death-ligand 1 (PD-L1), by peripheral blood mononuclear cells (PBMCs) is lacking. Here, high-dimensional mass cytometry (CyTOF) is used to investigate immune alterations and promising immunotherapeutic targets expression by PBMCs of CC patients. Results reveal that expression of EpCAM and PD-L1 on CD4^+^ T cells significantly increased in patients with CC, compared with age- and sex- matching healthy controls and patients with colonic polyps. These differences are also validated in an independent patient cohort using flow cytometry. Further analysis revealed that EpCAM^+^ CD4^+^ T cells are PD-L1^+^ CCR5^+^ CCR6^+^. Immunofluorescence staining results demonstrate that the increase of EpCAM^+^ CD4^+^ T cells is also observed in tumor tissues, rather than para-cancerous tissues. To ascertain the functional disorders of the identified cell subset, phosphorylated signaling protein levels are assessed using imaging mass cytometry. Increases in pp38 MAPK and pMAPKAPK2 are observable, indicating abnormal activation of pp38 MAPK-pMAPKAPK2 signaling pathway. Results in this study indicate that EpCAM^+^ CD4^+^ T cells may play a role in CC development. Detailed knowledge on the functionality of EpCAM^+^ CD4^+^ T cells is of high translational relevance.

## Introduction

Colon cancer (CC) is one of the main causes of cancer death worldwide (1, 2), with high incidence and mortality rates for all genders. Age-adjusted incidence rate is reported at 46.3 per 100,000 individuals per year worldwide, with only 12% 5-year survival rate for late-stage colorectal cancer patients (3). The prognosis of CC is generally unfavorable with high recurrence and metastasis rates. Immunotherapy, in combination with chemotherapy and anti-angiogenic agents, is gaining support as an effective approach to battle CC (4).

The epithelial cellular adhesion molecule (EpCAM), which overexpresses in epithelial cancer associated with enhanced malignant potential, is regarded as a desirable target for CC therapy (5). EpCAM-specific monoclonal antibodies have been used to treat human CC since the 1990s. Programmed death-1 (PD-1), an inhibitory receptor expressed on activated T cells, is regarded as a promising target protein for cancer therapy. Patient safety, clinical activity, and tolerability of PD-1 blockade were subsequently determined (6). However, the therapeutic efficacy of blocking these immune pathways is limited. Immunotherapeutic drugs targeting EpCAM have been developed by utilizing monoclonal antibodies (7, 8), bispecific antibodies (9), or conjugates with other agents, such as toxins (10). Data from clinical trials of EpCAM blockade suggest limited anti-tumor effects and low immune-activating efficacy. Reports of the therapeutic effects of PD-1 blockade in melanomas, non-small cell lung cancer, and renal-cell cancer patients are promising, however, exhibiting only a 3% treatment response rate with CC patients (11). Examining the role of EpCAM and PD-1 in carcinogenesis and malignant progression would aid the development of more efficacious immunotherapeutic schemes.

The importance of T cells in CC is well-established and fully illustrated by checkpoint blockade approaches (12, 13). Therefore, in addition to investigating the pool of functionally diverse T cell subsets, it is crucial to determine the expression levels of checkpoint-related molecules, such as EpCAM, PD-1, and programmed death-ligand 1 (PD-L1). Expression analyses of these checkpoints would help define the relative contributions of these molecules toward the immunotherapy responsiveness of CC patients. Although these suppressive markers have been widely investigated in tumor microenvironments, research on their expression by human immune cells and relevant importance in the peripheral blood remains scarce. Few studies characterize the importance of these molecules in circulating immune cells. Chevolet and colleges found that PD-L1 expression occurs in circulating cytotoxic T cells, which confers a worsen prognosis on overall survival rates (14). There is an urgent need for systemic and comprehensive profiling of immune checkpoint expressions.

Mass cytometry, also known as cytometry by time-of-flight (CyTOF), combines the high throughput of flow cytometry and the fine resolution of mass spectrometry (15, 16). In CyTOF, fluorophore reporters are substituted with rare metal isotope-conjugated antibodies (17, 18), circumventing the spectral overlap limitation in flow cytometric analysis and allows for a simultaneous determination of over 40 parameters at the single-cell level. Advances in data visualization and interpretation (19, 20) widens the application of CyTOF to various fields, including cellular reprogramming (21), phenotype heterogeneity mapping (22), and cell development analysis (23). Using this platform, we aim to investigate the checkpoints expression profile by specific T cell subsets and examine the underlying functional pathway mechanisms in these checkpoint expressed cell subsets (workflow presented in Supplementary Figure 1).

## Materials and Methods

### Subjects

This study is approved by the Ethical Committee and Institutional Review Board of Shanghai Jiao Tong University. Human peripheral blood specimens, obtained from patients diagnosed with either colon polyps or stage I-IV CC, were sampled from the First People's Hospital (Shanghai, China) between May 2016 and August 2018. Exclusion criteria included: (1) age over 70; (2) the presence of systemic disease or medication that could compromise the immune system; (3) having autoimmune disease or pregnancy; (4) having infectious disease, history of alcohol abuse, or other inflammatory conditions that could induce immunological impairments. Each patient was assigned to a stage based on the American Joint Committee on Cancer's (AJCC) staging system. Healthy subjects were recruited from the Medical Examination Center of the First People's Hospital. These individuals did not have a previous history or signs of increased blood pressure nor diabetes. In addition, they did not experience any fever within a defined period prior to the initiation of the study. All blood tests and imaging analyses, including computed tomography (CT) and X-rays were within normal ranges. Demographic characteristics are listed in Supplementary Table 1.

### PBMC Sample Collection, Pretreatment, and Preservation

Peripheral blood mononuclear cells (PBMCs) were obtained through a Ficoll-Paque density-gradient centrifugation of freshly drawn (within 4 h) EDTA-anticoagulated blood. After washing the extracted PBMCs once, the cells were stained with cisplatin for 5 min at 37°C to discriminate live-dead cells. Staining was terminated by adding cell-staining buffer (CSB) followed by centrifugation. Immediately after discarding the supernatants, the cells were fixed at room temperature in a paraformaldehyde solution at a final working concentration of 1.6% for 10 min. Cells were cryopreserved in DMSO-containing CSB at −80°C after washing.

### Antibody Staining and Data Acquisition by CyTOF

Cryopreserved PBMCs were thawed in a water bath pre-warmed to 37°C in 5 min. 3 × 10^6^ cells were then transferred into CSB for antibody staining. Mass cytometry antibody staining was implemented following previous study (24). The staining panel is listed in Supplementary Table 2. Stained cells were washed twice with 2 mL CSB, then incubated overnight at 4°C with 1 mL DNA Intercalator (Fluidigm, CA, USA), which was diluted in a Fix and Perm Buffer (Fluidigm) at a final concentration of 125 nM, which facilitated the discrimination between singlets, doublets, and triplets. Prior to data acquisition, cell pellets were resuspended in distilled water containing 10% EQ Four Element Calibration Beads (Fluidigm), and the cells were filtered through a 35-μm membrane. The cells were analyzed with Helios (Fluidigm) through several runs at an acquisition rate of ~500 events per second (25). Following the manufacturer's instructions, settings were on default.

### Data Processing and Analysis

The short-term signal fluctuations of Helios was normalized with EQ bead signals prior to data export and analysis (26). Abundance values obtained by mass cytometry were transformed using a scaled arcsinh with a factor of 5, which diminished near-zero noise values in the measurements. Surface marker expression in each channel was normalized based on the signal intensity at the 99.5th percentile across all samples, thus yielding expression values as *x*-fold of the 99.5th percentile values (27). To avoid debris and doublets, events recorded were gated based on DNA contents and cell length. Gating, viSNE plots (19), and spanning-tree progression analysis of density-normalized events (SPADE) analysis (28) were performed using the Cytobank platform (www.cytobank.org) as previously described (29). The following markers were used for SPADE and viSNE analysis: EpCAM, CD45RA, CCR5, CD4, CD8a, PD-L1, CD45RO, CCR6, PD-1, CD161, CTLA-4, CCR4, LAG3, CCR7, CD127, and CD25. Major cell subpopulations were annotated in the viSNE and SPADE maps based on prior knowledge of expected marker expression in various cell types. PhenoGraph clustering (27) was performed using all markers on a combined sample of 115,000 cells (subsampling of 5,000 cells per sample) using the cytofkit R package (30). A series of gates for T cell subpopulations (31) are presented in Supplementary Figure 2. Data plots and histograms were generated using MATLAB (MathWorks, R2018a).

### Flow Cytometry Validation

An independent cohort (*N* = 14, 7 CC patients, and 7 healthy controls) was included for validation (Supplementary Table 3). PE-conjugated EpCAM mAb (Invitrogen, CA, USA) and APC-conjugated CD4 mAb (Tonbo Biosciences, CA, USA) were purchased for flow cytometric analysis. Briefly, 100 μL freshly collected peripheral blood was blocked with Fc block (BioLegend, CA, USA) in the staining buffer for 10 min and incubated with antibodies for another 15 min at room temperature, then treated with red blood cell lysing buffer (BD Biosciences, CA, USA). The samples were washed with PBS and re-suspended in 300 μL PBS and analyzed with flow cytometry in a Canto II analyzer (BD Biosciences). The FlowJo X10 software (Treestar, CA, USA) was employed for data analysis. Cells were sequentially gated on lymphocytes (based on FFC vs. SSC), single cells (based on FSC-A vs. FSC-H), and CD4^+^ cells (indiscriminating live/dead) for the assessment of EpCAM expression.

### Immunofluorescence (IF) Staining

The tumor or para-cancerous tissues of the studied patients were sectioned by a pathologist who was blind to the patient group but aware of the study design. Sections were stained and examined under light microscope to assess the positivity for EpCAM (red) and CD4 (green). Ten regions of interests (ROIs) were randomly selected per sample. The average marker expression area (measured using ImageJ software) or cell counts of ten ROIs were used for subsequent statistical analysis.

### Imaging Mass Cytometry (IMC)

To assess the spatial distribution of EpCAM^+^ CD4^+^ T cell subpopulations in CC tissues, IMC (Hyperion, Fluidigm) (32) was employed to analyze tumor sections. Sections including para-cancerous and carcinoma tissues were collected and prepared as previously described (33). The antibody staining panel is listed in Supplementary Table 4. Image acquisition after daily tuning was carried out following manufacturer's instruction at a laser frequency of 200 Hz. Approximately 500 × 500 μm regions were selected based on bright field images. Two to five ROIs were selected per slide, which are dependent on the section size (Supplementary Table 5). The marker expression intensity associated with individual ROI were used as input for further analysis.

### Statistical Analysis

Data is expressed as mean ± standard deviation (SD) or as medians with interquartile ranges. Statistical significance between two groups was calculated using non-parametric Mann-Witney test. Rejection of the null hypothesis with a *p* value <0.05 was considered as significant.

## Results

### Expression Profiles of EpCAM, PD-1, and PD-L1 by T Cells

**Figure 1:**
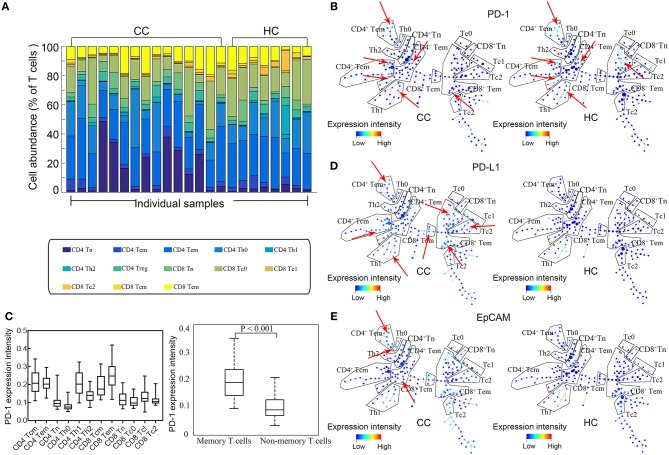
Expression profiles of EpCAM, PD-1, and PD-L1 by T cells. **(A)** Stacked bars of T cell subtypes in each individual sample. Cell abundance is expressed as percentage of total T cells. **(B)** Representative SPADE plot of PD-1 expression profile by T cells for colon cancer patients (CC) and healthy controls (HC). Color coding indicates the signified marker expression. Red arrows mark the extensive PD-1 expression by memory T cells. **(C)** Comparison of PD-1 in all T cell subtypes (left) and between memory and non-memory T cells (right). PD-1 is mainly expressed by memory T cells. **(D,E)** Representative SPADE plot of the expression profiles of PD-L1 **(D)** and EpCAM **(E)** by T cells. Color coding indicate the expression intensity of the corresponding markers. Red arrows indicate the abundant and heterogeneous patterns of PD-L1 and EpCAM expression in CC patients.

The eligibility of the volunteer CC patients and healthy controls (HCs) is screened as described in the Materials and Methods section. Only untreated patients that have a biopsy-proven diagnosis, along with clinical and pathologic assessments, are enrolled. Based on the combinations of surface markers, we simultaneously analyze expression of EpCAM, PD-1, and PD-L1 by CD4^+^/CD8^+^ naïve (Tn), CD4^+^/CD8^+^ central memory (Tcm), CD4^+^/CD8^+^ effective memory (Tem), subsets of CD4^+^ helper (Th0, Th1, Th2, & Treg), and CD8^+^ cytotoxic T cells (Tc0, Tc1, & Tc2). No significant difference is found between CC patients and HCs in the percentages of total T cells, CD4^+^ T cells, and CD8^+^ T cells (Figure 1A). In order to build a comprehensive landscape of immune blockade marker expressions, we employ the SPADE plot to sketch the profiles (Figure 1B).The results show that PD-1 is mainly expressed by memory T cells for both Tcm and Tem, irrespective of CD4^+^, and CD8^+^ (Figure 1C, left panel). Statistically significant difference in PD-1 expression is observable between memory and non-memory T cells (*P* < 0.001, Figure 1C, right panel). There is no difference in PD-1 expression by T cells between CC patients and HCs. PD-L1 expressions across all T cell subtypes are shown in Figure 1D and they exhibit increased expressions in CC patients. Figure 1E displays the representative SPADE plot of EpCAM expression landscape on T cell subsets.

### viSNE Analysis Shows an Increase of EpCAM^+^ CD4^+^ T Cells in CC Patients

**Figure 2:**
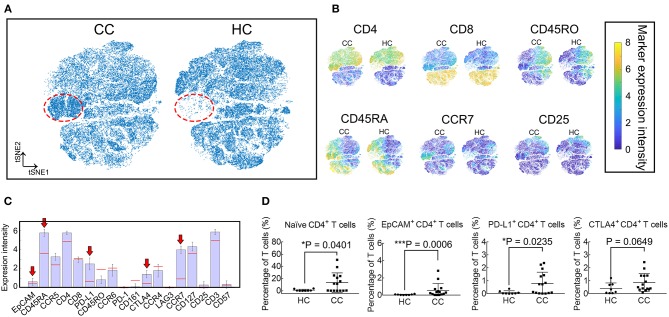
viSNE analysis revealed that EpCAM^+^ CD4^+^ T cells increased in CC patients. **(A)** Synthetic t-SNE maps depicting T cell subtypes in colon cancer patients (CC, *N* = 15) and healthy controls (HC, *N* = 8). Suspect populations that compose almost entirely of cells from CC patients are marked in the red dotted regions. **(B)** viSNE maps of T cells in CC and HC illustrating the expression of CD4, CD8, CD45RA, CD45RO, CCR7, and CD25. Color scale indicates the signified marker expression intensity. **(C)** Expression signatures of the identified subpopulations in **(A)**. Bar height indicates the median expression of each marker within a subpopulation. The horizontal line for each marker corresponds to median expression across all cells. Red arrows indicate positive immune blockade marker expressions. **(D)** Comparison of percentages in T cells of naïve (CD45RA^+^ and CCR7^+^), EpCAM^+^, PD-L1^+^, and CTLA4^+^ CD4^+^ T cells between CC and HC. Error bars indicate standard deviation (SD). Statistical differences are annotated with asterisk (^*^) and P (*p*-value) or ns, not significant. ^*^P < 0.05, ^**^P < 0.01, ^***^P < 0.001.

Given the high dimensionality of the mass cytometry data, we employ viSNE maps to visualize and search for high dimensional cell phenotypes that distinguish these groups (Figures 2A,B). A population, marked in the red dotted circles, is more abundant in CC patients. This population mainly contains CD4^+^ T cells expressing EpCAM, CD45RA, PD-L1, CTLA4, and CCR7 (Figure 2C). Results of viSNE analysis are reproduced with random event sampling (Supplementary Figures 3A,B), and PhenoGraph clustering is applied to the tSNE map (Supplementary Figure 3C). The subset marked in black polygon in Supplementary Figure 3B is subdivided into 4 clusters: S1, S8, S12, and S23. From the clustergram in Supplementary Figure 3D, we can tell that S12 and S23, two small clusters, are EpCAM^+^ CD4^+^ T cells, while S1 and S8 are EpCAM^−^ CD4^+^ Tn cells. Therefore, we further compare the frequencies of CD4^+^ Tn cells (CD45RA^+^ and CCR7^+^), PD-L1^+^ CD4^+^, EpCAM^+^ CD4^+^, and CTLA4^+^ CD4^+^ T cells between CC patients and HCs. CD4^+^ Tn cell increases are observable in CC patients (*P* = 0.0401, Figure 2D). Despite its low frequency, EpCAM and PD-L1 expression by CD4^+^ T cells are clinically relevant (*P* = 0.0006 and 0.0235, respectively, Figure 2D). CC patients had higher levels of EpCAM^+^ CD4^+^ T cells at the time of inclusion. No statistical difference is found in the percentages of CTLA4^+^ CD4^+^ T cells.

### Phenotypic Characterization of EpCAM^+^ CD4^+^ T Cells

**Figure 3:**
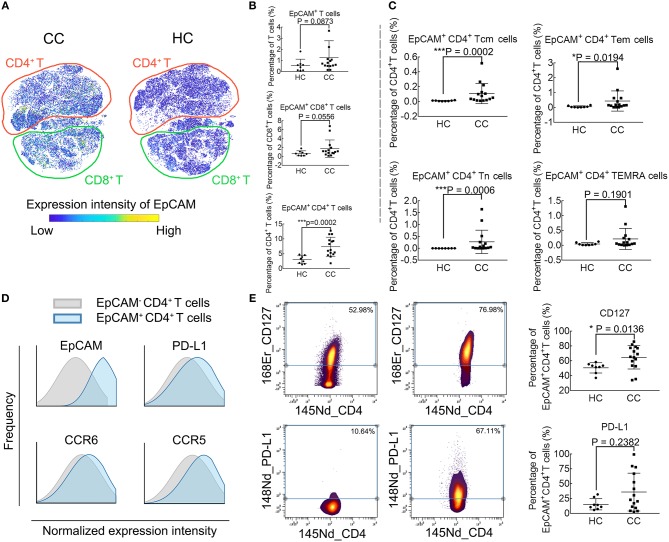
Phenotype profiling of EpCAM^+^ CD4^+^ T cells. **(A)** viSNE maps illustrating the expression profiles of EpCAM in T cell subsets in colon cancer patients (CC) and healthy controls (HC), with color-coding representing the expression intensity of EpCAM. The relative location of CD4^+^ (red) and CD8^+^ (cyan) T cells are marked through manual gating. **(B)** EpCAM expression in T cells and CD8^+^ T cells. **(C)** EpCAM expression in CD4^+^ T cell subpopulations. **(D)** Histograms comparing marker expression of PD-L1, CCR6, CCR5 in EpCAM^+^ and EpCAM^−^ CD4^+^ T cells. **(E)** Comparison of CD127 and PD-L1 expression in EpCAM^+^ CD4^+^ T cells in CC and HC groups. Statistically significant differences are annotated with asterisk (^*^) and *P* (*p*-value) or ns, not significant. ^*^P < 0.05, ^**^P < 0.01, ^***^P < 0.001.

Figure 3A demonstrates the EpCAM expression profile using the viSNE map. The EpCAM expression is prominent in CD4^+^ T cells, while no difference is observable in the relative levels of EpCAM^+^ CD8^+^ T cells or EpCAM^+^ T cells (Figure 3B). We further define whether differences exist among traditional CD4^+^ T cell subsets. There are significant differences in frequencies of EpCAM^+^ memory CD4^+^ T cells (both Tcm and Tem) and EpCAM^+^ CD4^+^ Tn cells between CC patients and HCs. However, this difference is not observable in the CD4^+^ TEMRA cells (CD45RA^+^ and CCR7^−^), as displayed in Figure 3C. To investigate other possible phenotypic alterations associated with EpCAM expression, we characterize EpCAM^+^ CD4^+^ T cells and compare the phenotype with EpCAM^−^ CD4^+^ T cells (Figure 3D). In contrast to EpCAM^−^ CD4^+^, EpCAM^+^ CD4^+^ T cells show higher levels of PD-L1, CCR5, and CCR6. We proceed to determine the functional marker expression intensities by EpCAM^+^ CD4^+^ T cells in CC patients, compared with HCs (Figure 3E). EpCAM^+^ CD4^+^ T cells exhibit higher levels of CD127 (Figure 3E, upper panel), the Interleukin-7 receptor (IL-7R), which might be involved in the maturation and aging of this subset. The expression of PD-L1 by EpCAM^+^ CD4^+^ T cells exhibits great heterogeneity in CC patients (Figure 3E, lower panel). The percentages of PD-L1^+^ EpCAM^+^ CD4^+^ T cells in EpCAM^+^ CD4^+^ T cells range from 2.38 to 99.39% in CC patients, and no significant difference is observed between CC and HC groups in the expression of PD-L1 by EpCAM^+^ CD4^+^ T cells.

### High Frequency of EpCAM^+^ CD4^+^ T Cell Subsets in the Tumor Microenvironment

**Figure 4:**
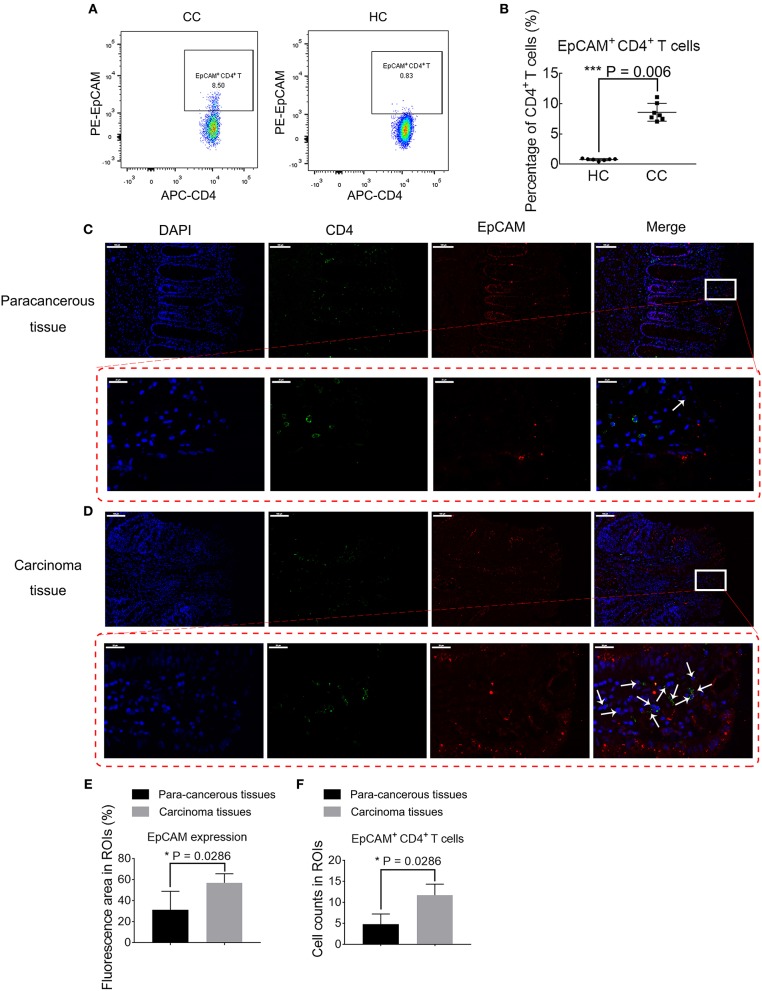
Validation of disparity in percentages of EpCAM^+^ CD4^+^ T cells. **(A)** Biaxial plots comparing percentages of EpCAM^+^ CD4^+^ T cells in colon cancer patients (CC) and healthy controls (HC). **(B)** Percentages of EpCAM^+^ CD4^+^ T cell subsets in PBMCs in CC and HC. The data is illustrated as the mean ± SD. **(C,D)** Immunofluorescence staining of para-cancerous **(C)** or carcinoma tissues **(D)** from CC. Data is representative result of four experiments. Scale bar = 100 μm. Lower panel in red dotted box represent magnified views of arrow-marked EpCAM^+^ CD4^+^ T cells in para-cancerous **(C)** or carcinoma tissues **(D)**. Scale bar = 20 μm. **(E)** Bar plot comparing the EpCAM expression area in carcinoma tissues and para-cancerous tissues. *N* = 4 for each group. Data is expressed as mean ± SD. **(F)** Bar plot depicting the differences in EpCAM^+^ CD4^+^ T cell abundance in carcinoma tissues and para-cancerous tissues. *N* = 4 for each group. Data is expressed as mean ± SD. Statistically significant differences are annotated with asterisk (^*^) and *P* (*p*-value) or ns, not significant. ^*^P < 0.05, ^**^P < 0.01, ^***^P < 0.001.

Differences of EpCAM expression by CD4^+^ T cells in CC patients and HCs are further validated using conventional flow cytometry in an independent, untreated patient cohort (Figures 4A,B). Patient cohort characteristics of this confirmation study are listed in Supplementary Table 3. Patients with CC have higher frequencies of EpCAM^+^ CD4^+^ T cells, as compared to HCs (*P* = 0.006, Figure 4B). Considering the higher expression of chemokines, including CCR5 and CCR6, which might attract EpCAM^+^ CD4^+^ T cells to the tumor sites, we determine whether EpCAM^+^ CD4^+^ T cells are present in the tumor microenvironment within CC patients using light microscopy. The results of IF staining (Figures 4C,D) show the EpCAM expression and CD4^+^ T cells distribution in tumor tissues and their para-cancerous tissues of CC patients. Magnified views with arrow-marked EpCAM^+^ CD4^+^ T cells in para-cancerous and carcinoma tissues are displayed in the lower panel of Figures 4C,D. EpCAM expression in tumor tissues is significantly higher than that in para-cancerous tissues (Figure 4E), which is in accord with former publications (34, 35). A higher number of EpCAM^+^ CD4^+^ T cells are observable in the carcinoma tissue images (Figure 4F). These results highlight a potential role of EpCAM^+^ CD4^+^ T cells in the course of CC development.

### Frequency of EpCAM^+^ CD4^+^ T Cells in Colonic Polyp Patients

**Figure 5:**
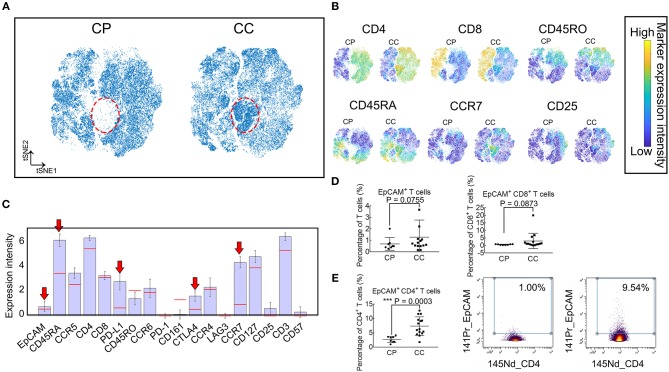
Frequency of EpCAM^+^ CD4^+^ T cells were elevated in PBMC of patients with CC, but not CP. **(A)** Synthetic t-SNE maps depicting T cell subsets in colonic polyp (CP, *N* = 8) and colon cancer (CC, *N* = 15) patient groups. Suspect populations that compose almost entirely of cells from CC patients are marked in the red dotted ellipse region. **(B)** viSNE maps of T cells in CP and CC illustrating the expression of CD4, CD8, CD45RA, CD45RO, CCR7, and CD25. Color coding indicates the signified marker expression intensity. **(C)** Expression signatures of the identified subpopulations in **(A)**. Bar height indicates the median expression of each marker within a subpopulation. The horizontal line for each marker corresponds to its median expression across all cells. Red arrow indicates three positive immune blockade marker expression. **(D)** Comparison of percentages in T cells of EpCAM^+^ and EpCAM^+^ CD8^+^ T cells between CP and CC. Error bar indicates standard deviation (SD). **(E)** Comparison of percentages of EpCAM^+^ CD4^+^ T cells in CD4^+^ T cells between CP and CC (left panel). Biaxial plots showing higher frequency of EpCAM^+^ CD4^+^ T cells in CC than in CP (right panel). Statistically significant differences are annotated with asterisk (^*^) and *P* (*p*-value) or ns, not significant. ^*^P < 0.05, ^**^P < 0.01, ^***^P < 0.001.

To verify whether the identified population EpCAM^+^ CD4^+^ T cells increased in patients with benign polyps, we examine a cohort of patients with colonic polyps (CP) and determine their T cell subset landscape using the same mass cytometry staining panel above (Supplementary Table 2). viSNE maps in Figures 5A,B show similar subpopulation deficiency pattern comparing CP and CC patients with the identified subset marked in red dotted circle. The phenotype characterization of the identified population is displayed in Figure 5C, showing similar expression pattern to the subsets identified in Figure 2. We further compare the frequencies of EpCAM^+^ T cells, EpCAM^+^ CD8^+^ T cells, and EpCAM^+^ CD4^+^ T cells between the CC and CP groups (Figures 5D,E). Only the EpCAM^+^ CD4^+^ T cells frequency is significantly different. To the best of our knowledge, this is the first documented study to demonstrate increased frequencies of EpCAM^+^ CD4^+^ T cells in the PBMC of CC patients, but not in CP patients or HCs.

### Activation of p38 MAPK Pathway in EpCAM^+^ CD4^+^ T Cells in the Tumor Microenvironment

**Figure 6:**
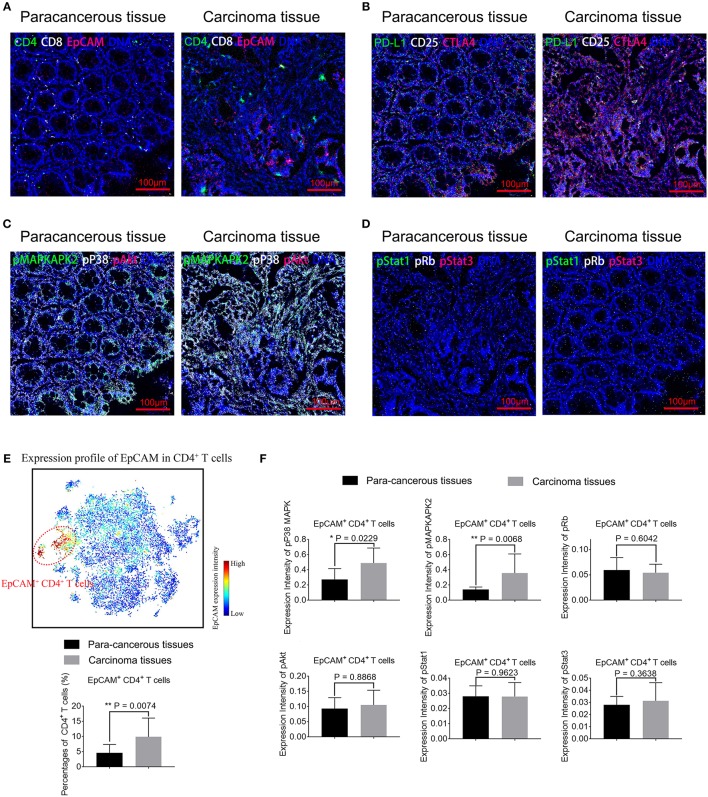
Representative mass cytometry images of para-cancerous and carcinoma tissue samples from colon cancer patients. EpCAM^+^ CD4^+^ T cells in carcinoma tissues display activated pp38 MAPK/pMAPKAPK2 pathway. **(A–D)** Representative IMC images of para-cancerous or tumor tissues from CC patients. Data is representative of results of three experiments. Scale bar = 100 μm. **(A)** Overlay of EpCAM (red), CD4 (green), CD8 (white), and DNA (blue). **(B)** Overlay of CTLA4 (red), PD-L1 (green), CD25 (white), and DNA (blue). **(C)** Overlay of pAkt (red), pMAPKAPK2 (green), pp38 MAPK (white), and DNA (blue). **(D)** Overlay of pStat3 (red), pStat1 (green), pRb (white), and DNA (blue). **(E)** viSNE map of cells extracted from IMC images (upper panel) with EpCAM^+^ CD4^+^ T cells marked in red dotted circles. Percentages of EpCAM^+^ CD4^+^ T cells in para-cancerous and tumor tissues are compared and displayed in the lower panel. **(F)** Expression of phosphorylated signaling proteins including pMAPKAPK2, pp38 MAPK, pAkt, pRb, pStat1, and pStat3 by EpCAM^+^ CD4^+^ T cells in para-cancerous and tumor tissues are compared. *N* = 11 for tumor tissue group and *n* = 9 for para-cancerous tissue group. The data is illustrated as the mean ± SD. Statistically significant differences are annotated with asterisk (^*^) and *P* (*p*-value) or ns, not significant. ^*^P < 0.05, ^**^P < 0.01, ^***^P < 0.001.

To assess the functional impact of EpCAM^+^ CD4^+^ T cells, we perform IMC on paired CC tissues and para-cancerous tissues. IMC utilizes laser ablation to generate particle plumes carried to time-of-flight detector via a stream of inert gas. IMC has a resolution comparable to light microscope and boasts simultaneous highly multiplex measurements through the use of isotopically labeled probes (33). Three tumor tissue samples and its corresponding para-cancerous tissues are obtained and stained for this experiment. In total, 29 markers are measured per cell in per slide, including markers that distinguish subpopulations and markers that characterize proliferative, signaling, and activation status (Supplementary Table 4). Two to five regions of interests were selected per slide (Supplementary Table 5). Figure 6A shows that predominantly EpCAM^+^ CD4^+^ T cells infiltrate is present in the tumor tissues, consistent with the results using IF. We also determine the distribution pattern of PD-L1, CD25, and CTLA4 in Figure 6B. Higher expression of PD-L1 by EpCAM^+^ CD4^+^ T cells is further validated. Furthermore, Figure 6C exhibits overlay images of expression profile of pp38 MAPK, pMAPKAPK2, pAkt, and DNA. Figure 6D displays overlay images of pRb, pStat1, pStat3, and DNA expression. We employ the tSNE algorithm to analyze the distributions and characteristics of cells extracted from these images and identify a subset of EpCAM^+^ CD4^+^ T cells marked in red dotted circle, in the upper panel in Figure 6E. Considering the small number of samples assessed by IMC, the cell percentages and marker expression intensity associated with individual ROI were used as input for statistical analysis. A significant increase of EpCAM^+^ CD4^+^ T cells is observed in tumor tissues. Increased expression of pp38 MAPK and pMAPKAPK2 by EpCAM^+^ CD4^+^ T cells is observed in the tumor tissues images, compared with para-cancerous tissues (*P* = 0.0229 and *P* = 0.0068, respectively, Figure 6F), suggesting that pp38 MAPK-pMAPKAPK2 signaling is up regulated. At the same time, no significant difference is detected in expression of pAkt, pRb, pStat1, and pStat3 by EpCAM^+^ CD4^+^ T cells between para-cancerous tissues and tumor tissues (Figure 6F), which suggests that complex upstream mechanisms might be involved in the development of CC.

## Discussion

Even though the immune checkpoints such as EpCAM and PD-1 are common targets for immunotherapy in various types of cancer, there is limited data recording their expression by human immune cells and relevant importance in the peripheral blood. In this study, we demonstrate, for the first time, the systemic and comprehensive expression profile of EpCAM, PD-1, and PD-L1 by T cells and their various subsets. Our results indicate the frequency of CD4^+^ Tn cells, PD-L1 levels, and EpCAM expressed by CD4^+^ T cells are higher in CC patients, in comparison to HCs. It is worth drawing attention to EpCAM^+^ CD4^+^ T cells since this subset increase only in CC patients but not in CP patients nor HCs. We characterize the phenotype of EpCAM^+^ CD4^+^ T cells as PD-L1^+^ CCR5^+^ CCR6^+^ and further confirm that this subset also increases in tumor microenvironments with irregulated p38 MAPK signaling pathway.

Previous studies demonstrate that a balanced loss and replacement of CD4^+^ Tn cells exists in the periphery during T cell maturation and differentiation (36, 37). Alterations in CD4^+^ Tn cells may likely affect T cell homeostasis and functionality. Previous study illustrates that Tn cells are prone to undergo apoptosis and lose quiescence in ovarian cancer patients, providing an insight into tumor-immune evasion mechanisms (38). Results in this study reveal that the frequency of CD4^+^ Tn cells is significantly higher in CC patients, in comparison to HCs, indicating compromised Tn cell priming and responses. However, the alterations in CD4^+^ Tn cells should be validated in an independent cohort study to further explore the underlying mechanisms.

With regard to PD-L1, Meniawy (39) and Arrieta (40) demonstrate higher proportions of PD-L1^+^ CD3^+^ T cells in patients with non-small cell lung cancer (NSCLC) and a correlation between PD-L1 expression on peripheral T cells and clinical outcomes in EGFR-TKI-treated NSCLC. Results in this study, to some extent, corroborate, and expand the findings of previous works. We demonstrate that PD-L1 is expressed by multiple circulating T cell subsets. Significant differences are found in frequencies of PD-L1^+^ CD4^+^ T cells, but not in PD-L1^+^ CD3^+^ T cells in CC patients, compared to HCs. Further studies should focus on the prognostic value of assessing the expression of PD-1 and PD-L1 on the surface of peripheral T cells in CC patients that are receiving PD-1 blockade treatments.

EpCAM, a 40-kDa transmembrane glycoprotein, is a promising therapeutic target for antitumor schemes for its tumor-specific overexpression (41, 42). To the best of our knowledge, this paper is the first investigative report of EpCAM expression on circulating immunocytes and the first study to reveal the phenotypic characteristics and the intracellular signaling protein expression profile of EpCAM^+^ CD4^+^ T cells. EpCAM expression is demonstrated to correlate with proliferation and low differentiation (43). Recent research confirms that therapeutic strategies, using EpCAM/CD3-bispecific antibody, are significantly effective in tumor elimination (44). One of the explanation might be that the bi-functional antibody has the ability to bind EpCAM expressing cancer cells as well as cytotoxic T cells (45). Here, we propose that this treatment might influence the compositions or functions of EpCAM^+^ CD4^+^ T cells. These are promising prospects to investigate the effect of EpCAM/CD4-bispecific antibody, as part of combination strategies with other immunotherapies, for CC patients.

In this study, we further characterize EpCAM^+^ CD4^+^ T cells as PD-L1^+^ CCR5^+^ CCR6^+^. It is widely accepted that ligation of PD-1 and PD-L1 leads to T cell dysfunction, exhaustion, and tolerance (8). High expression of PD-L1 by EpCAM^+^ CD4^+^ T cells indicates inhibited antitumor function or ineffective immune response. Expressions of chemokines, including CCR5 and CCR6, suggest that EpCAM^+^ CD4^+^ T cells are directed to the tumor sites, which is corroborated in this study through IF and IMC, as shown in Figures 4C, 6A. All these results show that EpCAM^+^ CD4^+^ T cells play an immune suppressive role in CC.

Many researchers explore the role of MAPK signal pathways in EpCAM^+^ cells (11, 46). The p38 MAPK, one serine/threonine kinases of the MAPK family [namely ERK, p38 MAPK, and c-junctional N-terminal kinases (JNK)], play a central role in long-term self-renewal, survival growth, differentiation, and apoptosis (47). Activated p38 MAPK can phosphorylate and activate MAPKAPK2 and is associated with tumor initiation and development (9). We reveal excessively activated pp38 MAPK-pMAPKAPK2 signaling expressed by EpCAM^+^ CD4^+^ T cells in tumor tissues in CC patients, indicating that the p38 MAPK pathway might be a potential therapeutic target in EpCAM^+^ CD4^+^ T cell-rich CC patients. These findings should be further validated, however, considering the actual number assessed by IMC was small and that the functional investigation was preliminary and exploratory.

Future work could concentrate on clinical responses and prognosis prediction values of proportions and functions of EpCAM^+^ CD4^+^ T cells, as well as the therapeutic values of this subset. Further understanding of the role of EpCAM in the regulation of immune functions in any given subtype of immunocytes is in great demand.

After stringent exclusion criteria, patient groups in this study are homogeneous, treatment-naïve, and compatible with statistical requirements. Yet, the results here require prospective validation in an independent and larger patient cohort, together with the identification of the mechanisms underlying the observable increase of EpCAM^+^ CD4^+^ T cells. Nonetheless, this pilot study demonstrates the EpCAM^+^ CD4^+^ T cells abnormal in CC patients and provides an insight in immunotherapy decisions and prognosis for CC patients.

## Data Availability

Mass cytometry and conventional flow cytometry data in the form of FCS files can be accessed from FlowRepository (http://flowrepository.org/) via accession number FR-FCM-Z24L.

## Ethics Statement

The study was approved by the Ethical Committee and Institutional Review Board of Shanghai Jiao Tong University. All participants provided written informed consent.

## Author Contributions

TZ and JL: conceptualization and methodology. TZ, ZT, and BW: analysis. TZ, YL, HJ, and AW: manuscript writing. HL and XD: resources and supervision.

### Conflict of Interest Statement

The authors declare that the research was conducted in the absence of any commercial or financial relationships that could be construed as a potential conflict of interest.
