# Supplementary material for: Immunocyte Profiling Using Single-Cell Mass Cytometry Reveals EpCAM+ CD4+ T Cells Abnormal in Colon Cancer
Source: Front Immunol. 2019 Jul 9;10:1571. doi: 10.3389/fimmu.2019.01571 (PMC6629930; doi:10.3389/fimmu.2019.01571)
Supplement: Supplementary file 1 [file Data_Sheet_1.docx]

***Supplementary Material***

**Supplementary Figures and Tables**

**Supplementary Tables**

**Supplementary Table S1. Demographic characteristics of CyTOF datasets.**

| Groups  N  Age (years)  Sex (F/M)  WBC (x10^9^/ml) | HC  8  52.75 ± 7.91  2/6  6.54 ± 0.73 | CC  15  59.50 ± 7.62  6/9  7.95 ± 2.49 | CP |
| --- | --- | --- | --- |
|  |  |  | 8 |
|  |  |  | 52.00 ± 9.91 |
|  |  |  | 3/5 |
|  |  |  | 6.28 ± 1.27 |
| Note:  HC: healthy control;  CC: colon cancer patients;  CP: colonic polyp patients;  WBC: white blood cells;  Data is expressed as mean ± SD. | | |  |

**Supplementary Table S2. CyTOF antibody staining panel.** The antibody panel was designed to obtain a comprehensive view of T cell subtype heterogeneity. For this purpose, the panel included markers that distinguish major T cell subtypes, e.g., CD4^+^ and CD8^+^ T cells. In addition, markers were included that allowed the identification of phenotypically distinct subsets within those immune cell subtypes, e.g., naïve (CD45RA^+^) and memory (CD45RO^+^) T cells.

| Antigen | Mass Tag | Clone | Vendor | Cat# |
| --- | --- | --- | --- | --- |
| CD45 | ^89^Y | HI30 | Fluidigm | 3089003B |
| EpCAM/CD326 | ^141^Pr | 9C4 | Fluidigm | 3141006B |
| CD45RA | ^143^Nd | HI100 | Fluidigm | 3143006B |
| CCR5/CD195 | ^144^Nd | NP-6G4 | Fluidigm | 3144007A |
| CD4 | ^145^Nd | RPA-T4 | Fluidigm | 3145001B |
| CD8a | ^146^Nd | RPA-T8 | Fluidigm | 3146001B |
| PD-L1/CD274 | ^148^Nd | 29E.2A3 | Fluidigm | 3148017B |
| CD45RO | ^149^Sm | UCHL 1 | Fluidigm | 3149001B |
| CCR6/CD196 | ^151^Eu | G034E3 | Fluidigm | 353427 |
| PD-1/CD279 | ^155^Gd | EH12.2H7 | Fluidigm | 3155009B |
| CD161 | ^160^Gd | HP-3G10 | BioLegend | 339902 |
| CTLA_4/CD152 | ^161^Dy | 14D3 | Fluidigm | 3161004B |
| CCR4/CD194 | ^163^Dy | L291H4 | BioLegend | 359402 |
| LAG3/CD223 | ^165^Ho | 874501 | Fluidigm | 3165028B |
| CCR7/CD197 | ^167^Er | G043H7 | Fluidigm | 3167009A |
| CD127 | ^168^Er | A019D5 | Fluidigm | 3168017B |
| CD25 | ^169^Tm | 2A3 | Fluidigm | 3169003B |
| CD3 | ^170^Er | SP34-2 | Fluidigm | 3170007B |
| CD57 | ^172^Yb | HCD57 | Fluidigm | 3172009B |
| Intercalator | ^193^Ir | -- | Fluidigm | 201192B |
| Cisplatin | ^195^Pt | -- | Sigma-Aldrich | 479306 |

**Supplementary Table S3. Demographic characteristics of flow cytometry datasets.**

| Groups  N  Age (years)  Sex (F/M) | HC  7  63.6 ± 3.30  4/3 | CC  7  61.87 ± 2.23  2/5 |
| --- | --- | --- |
| Note:  HC: healthy control;  CC: colon cancer patients;  Data are expressed as mean ± SD. | | |

**Supplementary Table S4. Imaging mass cytometry antibody staining panel.** The antibody panel was designed to obtain the dysregulated signaling information of T cells subtypes.

| Antigen | Mass Tag | Clone | Vendor | Cat# |
| --- | --- | --- | --- | --- |
| EpCAM/CD326 | ^141^Pr | 9C4 | Fluidigm | 3141006B |
| CD19 | ^142^Nd | HIB19 | Fluidigm | 3142001B |
| CD45RA | ^143^Nd | HI100 | Fluidigm | 3143006B |
| CCR5/CD195 | ^144^Nd | NP-6G4 | Fluidigm | 3144007A |
| CD4 | ^145^Nd | RPA-T4 | Fluidigm | 3145001B |
| CD8a | ^146^Nd | RPA-T8 | Fluidigm | 3146001B |
| CD11c | ^147^Sm | 3.9 | Abcam | ab11029 |
| PD-L1/CD274 | ^148^Nd | 29E.2A3 | Fluidigm | 3148017B |
| CD45RO | ^149^Sm | UCHL 1 | Fluidigm | 3149001B |
| pRb(Ser807/811) | ^150^Nd | J112-906 | Fluidigm | 3150013A |
| CD14 | ^151^Eu | M5E2 | Fluidigm | 3151009B |
| pAkt(pS473) | ^152^Sm | D9E | Fluidigm | 3152005A |
| pStat1(pY701) | ^153^Eu | 4a | Fluidigm | 3153005A |
| CD56 | ^154^Sm | RNL-1 | Abcam | ab9108 |
| PD-1/CD279 | ^155^Gd | EH12.2H7 | Fluidigm | 3155009B |
| pP38(180/182) | ^156^Gd | D3F9 | Fluidigm | 3156002A |
| pStat3(Y705) | ^158^Gd | 4/P-STAT3 | Fluidigm | 3158005A |
| pMAPKAPK2(Thr334) | ^159^Tb | 27B7 | Fluidigm | 3159010A |
| CTLA_4/CD152 | ^161^Dy | 14D3 | Fluidigm | 3161004B |
| LAG3/CD223 | ^165^Ho | 874501 | Fluidigm | 3165028B |
| CCR7/CD197 | ^167^Er | G043H7 | Fluidigm | 3167009A |
| CD33 | ^166^Er | WM53 | Abcam | ab30371 |
| CD127 | ^168^Er | A019D5 | Fluidigm | 3168017B |
| CD25 | ^169^Tm | 2A3 | Fluidigm | 3169003B |
| CD3 | ^170^Er | SP34-2 | Fluidigm | 3170007B |
| pErk1/2(202/204) | ^171^Yb | D13.14.4E | Fluidigm | 3171010A |
| HLA-DR | ^173^Yb | L243 | Fluidigm | 3173005B |
| CD11b | ^209^Bi | ICRF44 | Fluidigm | 3209003B |
| CD45 | ^89^Y | HI30 | Fluidigm | 3089003B |
| Intercalator | 193Ir | -- | Fluidigm | 201192B |

**Supplementary Table S5. Numbers of regions of interests (ROI) acquired by IMC for each sample.** C, short for carcinoma tissue; P, short for para-cancerous tissue.

| Sample ID | Number of ROIs |
| --- | --- |
| 1317-C | 3 |
| 1317-P | 5 |
| 1466-C | 4 |
| 1466-P | 2 |
| 1468-C | 4 |
| 1468-P | 2 |

**Supplementary Figures**


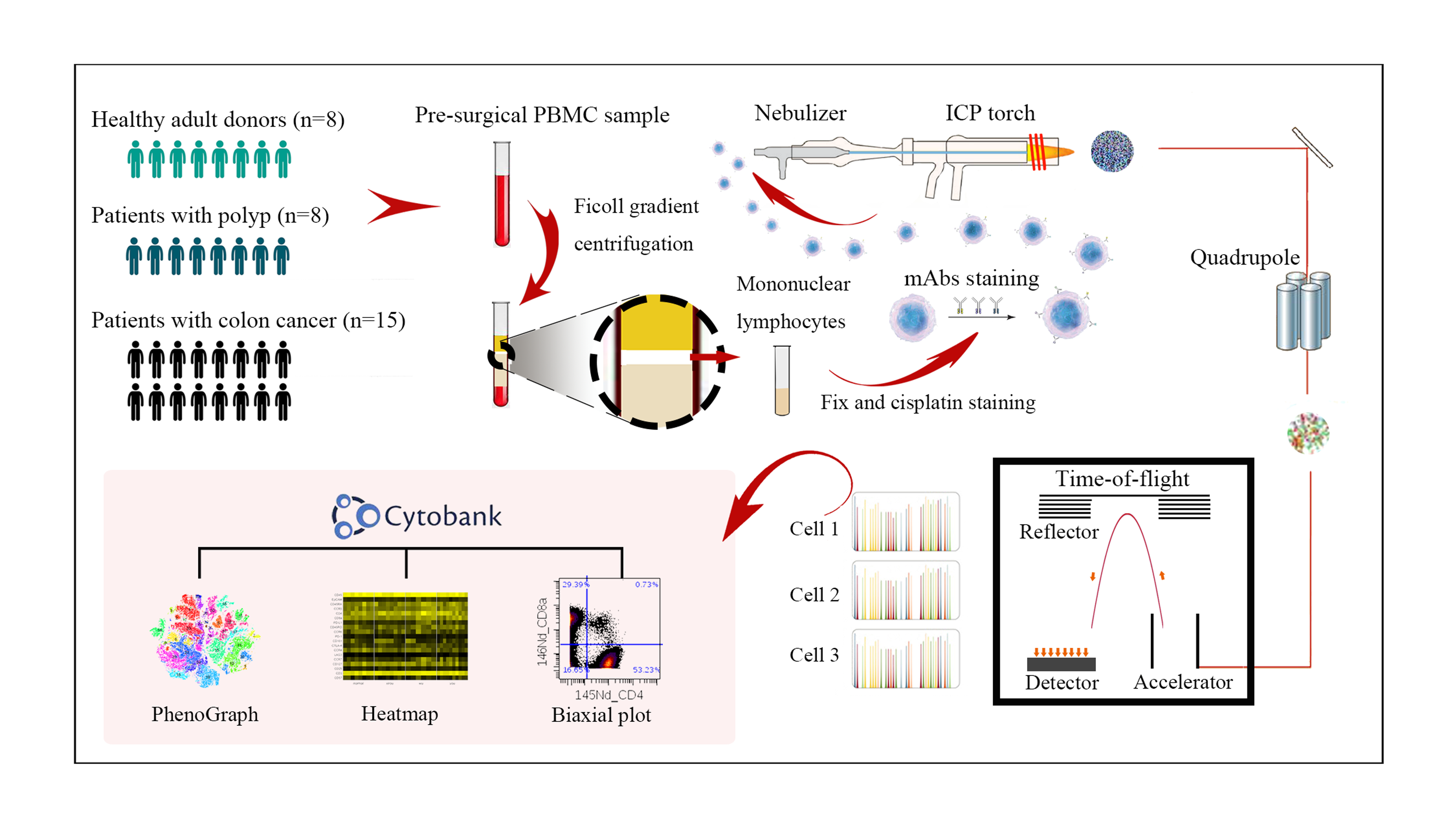


**Supplementary Figure S1. Workflow of experimental design.** Whole blood samples from 8 healthy volunteers as control, 8 patients with colonic polyps, and 15 patients with colon cancer were collected. Peripheral blood mononuclear cells were isolated and stained with a panel of 19 antibodies (Supplementary Table S2). Data was acquired using mass cytometry and analyzed as described in the Materials and Methods section.


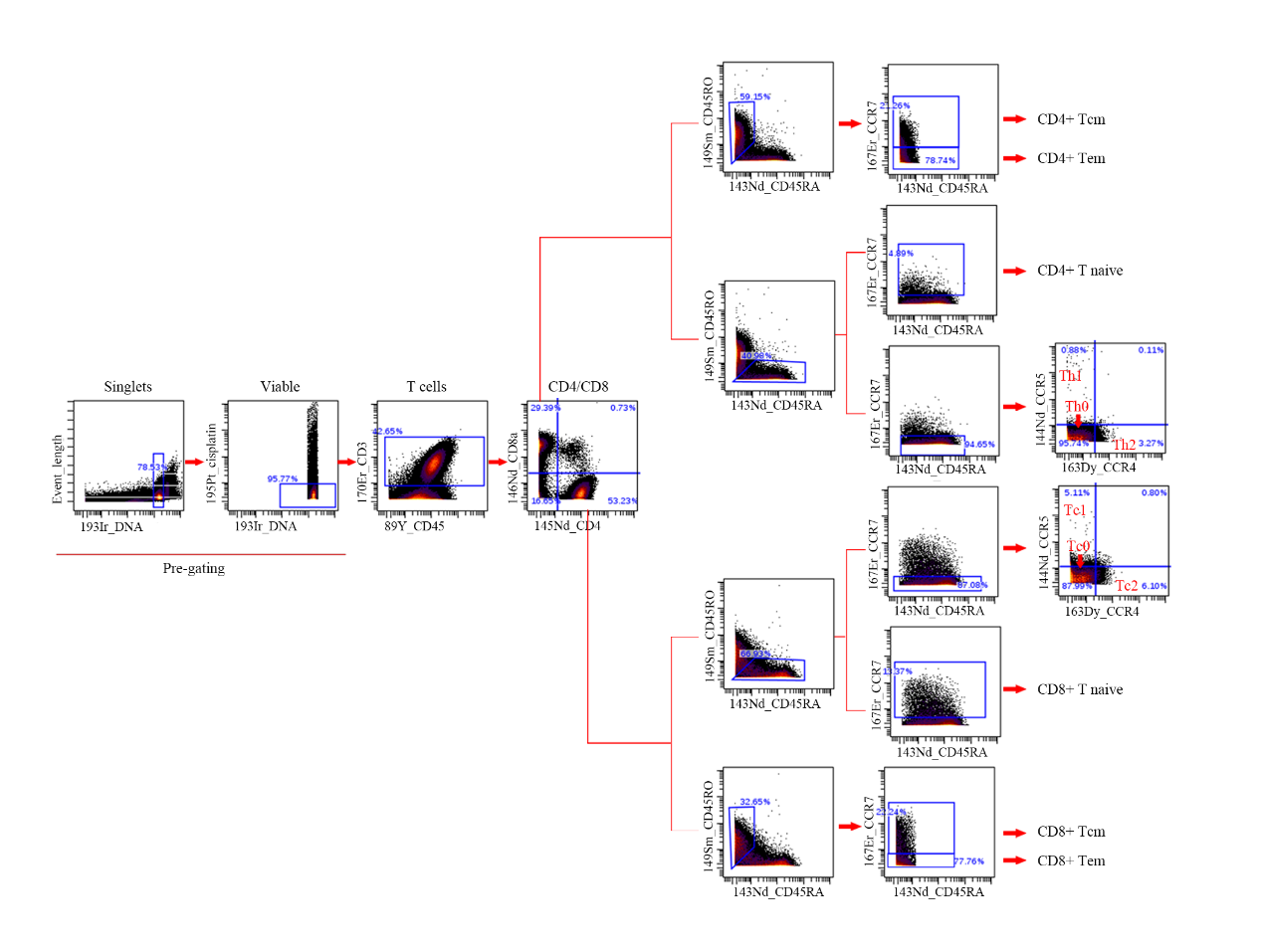


**Supplementary Figure S2.** **Gating hierarchy scheme.** Typical biaxial gating hierarchy strategy was adopted to define classical T cell subpopulations. Ten markers were combined to define thirteen cell types. Pre-gating step involved removal of doublets, dead cells, and cell debris.





**Supplementary Figure S3. PhenoGraph clustering analysis. (A)** tSNE landscape was created to cross-validate the results in Figure 2. The map is colored according to sample groups. (**B**) tSNE maps with color representing expression of indicated markers on cells. (**C**) The same tSNE map presented in (**A**), labeled by PhenoGraph classification results. Color coding indicates cluster distribution. (**D**) Hierarchical clustering of expression pattern on the PhenoGraph identified clusters.
